# Exceptional soft tissues preservation in a mummified frog-eating Eocene salamander

**DOI:** 10.7717/peerj.3861

**Published:** 2017-10-03

**Authors:** Jérémy Tissier, Jean-Claude Rage, Michel Laurin

**Affiliations:** 1 Cenozoic Research Group, JURASSICA Museum, Porrentruy, Switzerland; 2 Department of Geosciences, University of Fribourg, Fribourg, Switzerland; 3 Département Histoire de la Terre, UMR 7207, Centre de Recherches sur la Paléobiodiversité et les Paléoenvironnements, CNRS/MNHN/UPMC (Sorbonne Universités), Museum national d’Histoire naturelle, Paris, France

**Keywords:** Soft tissues, Phosphorites du Quercy, Exceptional preservation, Extinct urodele, Ecology, Synchrotron microtomography, Three-dimensional preservation, Eocene

## Abstract

Fossils are almost always represented by hard tissues but we present here the exceptional case of a three-dimensionally preserved specimen that was ‘mummified’ (likely between 40 and 34 million years ago) in a terrestrial karstic environment. This fossil is the incomplete body of a salamander, *Phosphotriton sigei*, whose skeleton and external morphology are well preserved, as revealed by phase-contrast synchrotron X-ray microtomography. In addition, internal structures composed of soft tissues preserved in three dimensions are now identified: a lung, the spinal cord, a lumbosacral plexus, the digestive tract, muscles and urogenital organs that may be cloacal glands. These are among the oldest known cases of three-dimensional preservation of these organs in vertebrates and shed light on the ecology of this salamander. Indeed, the digestive tract contains remains of a frog, which represents the only known case of an extinct salamander that fed on a frog, an extremely rare type of predation in extant salamanders. These new data improve our scarce knowledge on soft tissue anatomy of early urodeles and should prove useful for future biologists and palaeontologists working on urodele evolutionary biology. We also suggest that the presence of bat guano and carcasses represented a close source of phosphorus, favouring preservation of soft tissues. Bone microanatomy indicates that *P. sigei* was likely amphibious or terrestrial, and was probably not neotenic.

## Introduction

The ‘Phosphorites du Quercy’, in southwestern France, include numerous karstic fissures in-filled by phosphatic sediments rich in vertebrate remains (Legendre et al., 1997; Pélissié & Sigé, 2006). Almost all remains appear as classical disarticulated fossil bones, but a few of them (a salamander, anurans and snakes) are spectacular cases of exceptional preservation; the animals are entirely mineralized, including the skin, in three dimensions. Unfortunately, these ‘mummies’ were collected in the 19th century and their precise provenance and geological age are unknown. However, it is suspected that they come from the late middle or late Eocene (Laloy et al., 2013; Tissier et al., 2016).

**Figure 1:**
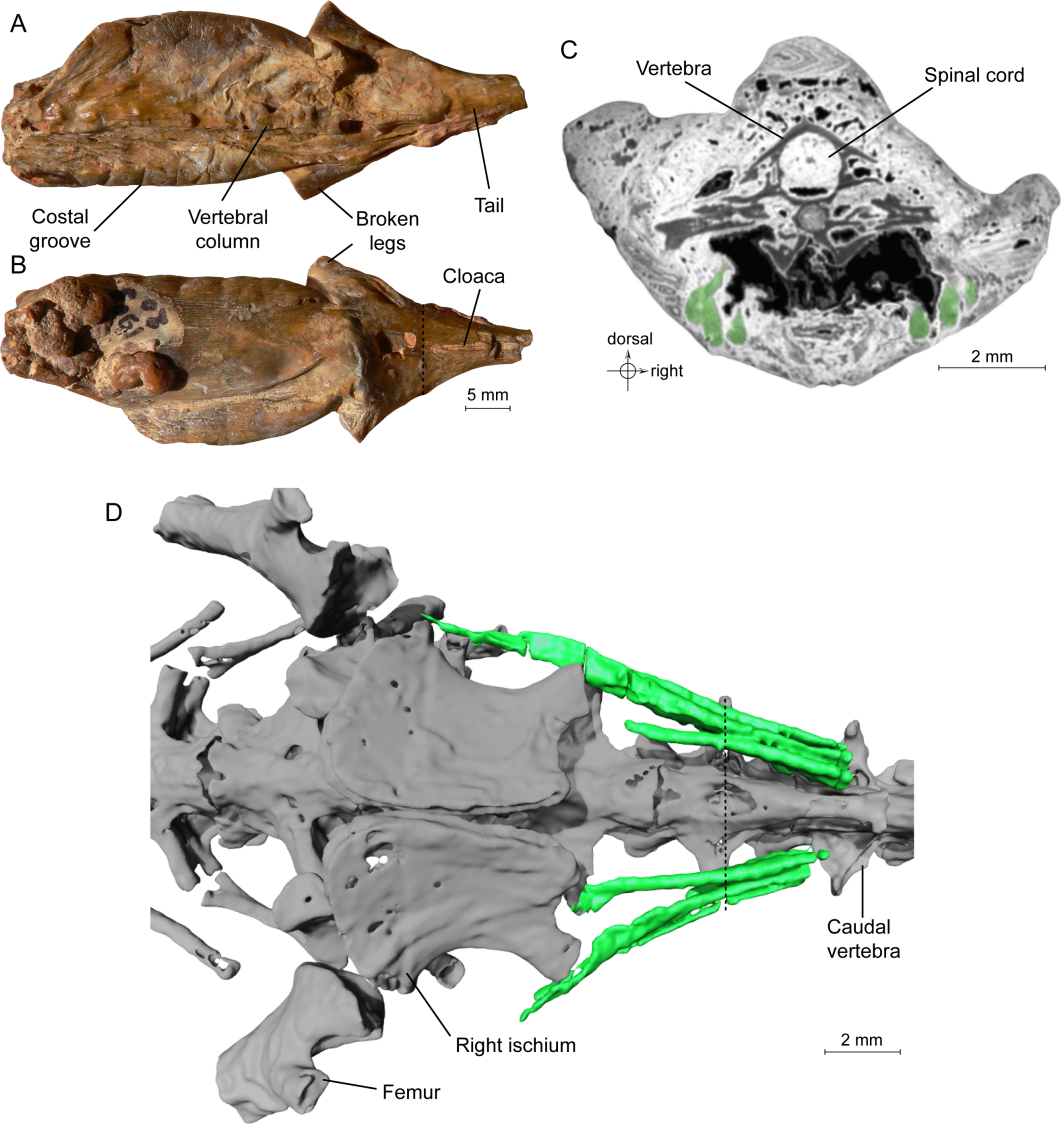
Specimen MNHN.F.QU17755, holotype of *Phosphotriton sigei*. (A and B) Fossil in dorsal and ventral views. Some characteristics of urodeles, such as costal grooves or scaleless skin, are observable on the external aspect of the specimen. The cloaca and vertebral column are visible. The dotted line represents the position of the tomogram illustrated in Fig. 1C. (C) Tomogram of the tail part of the animal showing the muscles, in green, ventral and lateral to the vertebrae, and the spinal cord preserved inside the neural canal of a vertebra. Bony material is characterized by a dark grey shade, because of its light density, compared to the mineral matrix (grey or white) and void (black). Soft-tissues are also mostly darker than the mineral matrix, but are mainly recognizable by their structure and shape, on tomograms or in 3D. (D) 3D reconstruction of undetermined tail muscles, in green, which could attach to the ischium or femur. Dotted line represents the position of the tomogram illustrated in Fig. 1C.

Until recently, only the external morphology of the ‘mummies’ was known. However, recent tomographic studies showed that the skeleton is preserved within the ‘mummies’ of the frog *Thaumastosaurus gezei* (Laloy et al., 2013) and of the salamander *Phosphotriton sigei*) (Tissier et al., 2016). The specimen of *P. sigei* includes a large part of the trunk (preserved posterior to the shoulder girdle), the anterior portion of the tail and the proximal portions of the hind limbs (Fig. 1A). The right side of the trunk is crushed. Diagnostic external features include the absence of scales, the presence of costal grooves visible on the left side, and the presence of a longitudinally slit-shaped cloaca.

The study of the skeleton of *Phosphotriton* confirmed that this fossil is a urodele amphibian; more precisely, the phylogenetic analysis presented by Tissier et al. (2016) suggested that it is a stem-salamandrid, although they did not definitely discard relationships with the Plethodontidae.

The microtomography of *Phosphotriton* clearly suggested also that, in addition to the skeleton, soft tissues were preserved. Subsequent segmentations indeed displayed various soft tissues within this specimen, which are the subject of the present article. We show here that the observed organs are not infills of cavities but are really the organs themselves that were permineralized.

## Materials and Methods

The only specimen of *P. sigei* (MNHN.F.QU17755) was investigated with the help of propagation phase contrast synchrotron X-ray microtomography, which gives a better contrast to differentiate tissues from the mineral matrix than traditional absorption based synchrotron X-ray microtomography. The method and parameters of acquisition are described in Tissier et al. (2016). A 3D model is given in Supplemental Information 1 in 3D PDF file format.

Several structures composed of soft tissues are preserved and may be identified on the tomograms. They can be distinguished from bones and mineral matrix by their shape, density on tomograms and structure. Their identification is based on comparisons with the literature on urodele soft anatomy because dissecting extant specimens would not have added to what may be drawn from the available literature. Therefore, we use their position in the body, their shape in three dimensions, and their internal structure on tomograms to identify them based on comparisons with existing descriptions. Some of them remain difficult to identify precisely, for several reasons (incompleteness of the organ, segmentation difficulties, small size, etc.). Proposed identifications are therefore tentative in some cases (e.g., an organ of the uro-genital system), although some appear to be certain (spinal cord, lumbosacral plexus).

To assess the lifestyle of *P. sigei*, we analysed the compactness profile of femoral mid-diaphyseal virtual cross-sections. We then used these data to infer the lifestyle with the inference models published by Laurin, Canoville & Quilhac (2009). These are based on statistical analyses of femoral compactness profiles of 46 extant urodele species. Variables in the models were selected through backward elimination and forward selection procedures, respectively, which led to two models with different combinations of variables.

## Results

**Muscles**. Not all muscles appear to have been fossilized. In addition, most muscles were not segmented, because of their irregular, ill-defined contour; their segmentation would have required too much subjective interpretation and would have been very time-consuming. It has not been possible to precisely identify the preserved muscles, as their position in the specimen is not sufficient for this. Only three of them were segmented: they are recognizable by their fibrous structure and shape (Figs. 1C–1D). We suppose that these may be three ventral caudal muscles described by Francis (1934: 102–103), which arise from the fourth caudal vertebra (i.e., M. caudali-pubo-ischio-tibialis, M. ischio-caudalis (the most mesial one, which inserts on the posterior border of the ischium) and M. caudalifemoralis (the most lateral one, which inserts on the femur)). Francis (1934) described them as having an ‘oval cross-section’, and being ‘narrow and strap-like’, which fits the muscles disclosed here. Their function is to flex the tail.

**Figure 2:**
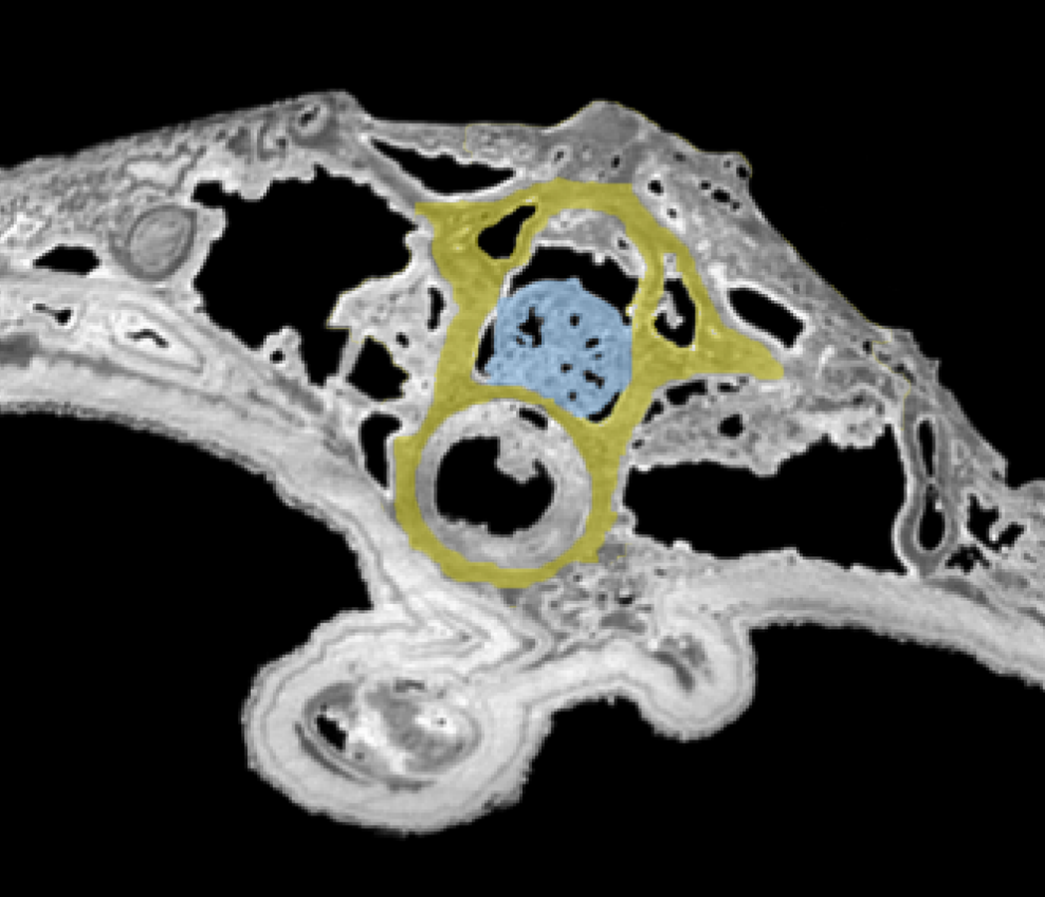
Tomogram of the trunk portion of the specimen MNHN.F.QU17755. Spinal cord is in blue, within the neural canal of a trunk vertebra (in yellow).

**Spinal cord**. It is preserved and visible in section in some vertebrae, inside the neural canal (Fig. 1C, 2). In the vertebrae where it is not preserved, only an empty space is visible (black on the tomogram). Unfortunately, that organ could not be segmented because its preservation is too uneven. No bony support of the spinal cord is visible. Spinal cord supports are bony processes that extend in the neural canal of vertebrae (Wake & Lawson, 1973; Skutschas, 2009; Skutschas & Baleeva, 2012). The fact that supports do not appear on the images does not necessarily mean that they were absent. These structures, which occur in various salamanders, are tiny and difficult to detect on tomograms (Skutschas & Baleeva, 2012; PP Skutschas, pers. comm., 2015).

It seems clear that in this specimen, the soft tissues are mineralized, even internally, and do not represent cavity filling. Indeed, the structure of the spinal cord is in some rare places well preserved, in three dimensions. Notably, the external surface of the cord is bordered by empty space on tomograms (Fig. 2), which would not happen if this was a case of cavity filling preservation. This ‘empty space’ was originally occupied by the cerebrospinal fluid, which cannot fossilize. Internal structure is difficult to discern but it is nevertheless reminiscent to what can be observed in extant urodeles, with a central canal (see Davis, Duffy & Simpson, 1989: fig. 6A for example).

**Figure 3:**
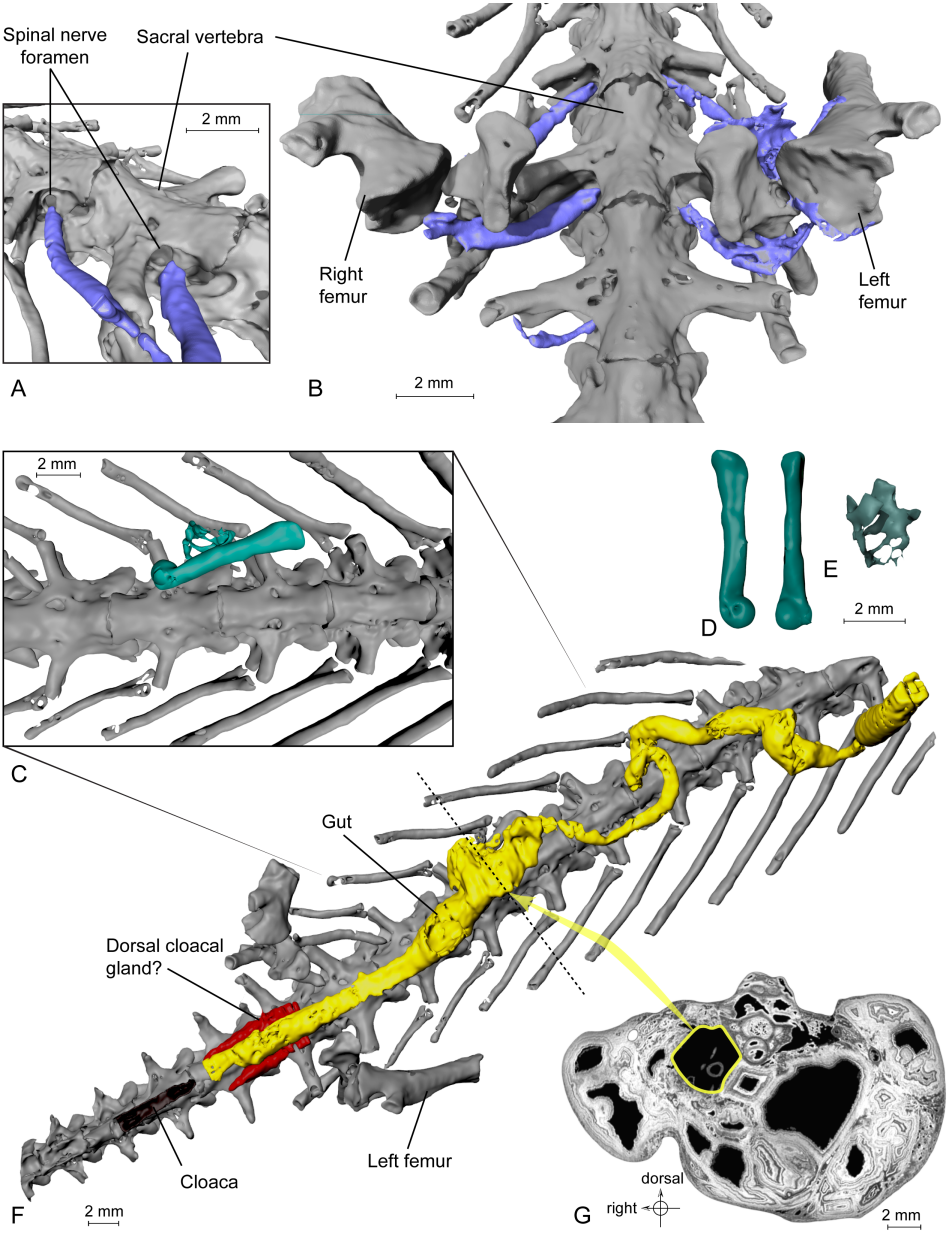
Exceptional preservation of nerves, digestive tract and stomachal content. (A and B) 3D reconstructions of the pelvic section of MNHN.F.QU17755, in laterodorsal (A) and ventral (B) views. The lumbosacral plexus (in blue) is partly preserved. Nerves exit the last trunk, the sacral and the first caudosacral vertebrae through the spinal nerve foramina. (C) Preserved bones of an anuran frog (ranoid?), in green, inside the digestive tract (not shown, to better reveal its content; see Fig. 3F) of MNHN.F.QU17755. (D) Anuran humerus found inside digestive tract of MNHN.F.QU17755, in lateral and ventral views. (E) Anuran vertebrae found inside digestive tract of MNHN.F.QU17755. The centrum is very thin; the holes may represent segmentation artifacts. (F) 3D reconstruction of MNHN.F.QU17755 in ventral view, showing the nearly complete digestive tract. The caudal end is very close to the cloaca, and is bordered near the pelvic girdle by presumed dorsal cloacal glands (see Fig. 4A). The dotted line represents the position of the virtual section illustrated in Fig. 3G. (G) Virtual section of the trunk, showing the digestive tract (in yellow) and its content (frog bones).

**Lumbosacral plexus**. This plexus comprises three nerves that emerge from the spinal cord through the spinal foramina of the last trunk vertebra, the sacral vertebra and the first caudosacral vertebra. These spinal foramina are large (Tissier et al., 2016: figs. 5B and 6B–6C). These three nerves merged lateral to the ilia to form the lumbosacral plexus (Figs. 3A–3B) and the resulting nerve entered the hind limb; this is similar to the disposition observed in *Necturus* by Wischnitzer (1979). The nerve exiting the last trunk vertebra corresponds to the ‘sixteenth spinal nerve’ in *Salamandra* Francis (1934: 173). The middle nerve of the plexus, emerging from the sacral vertebra, is the thickest, correlatively with the size of the foramen. It is termed ‘seventeenth spinal nerve’ in *Salamandra* by Francis (1934). The nerve exiting from the first caudosacral vertebra, called *nervus spinalis 18* in *Salamandra* (Francis, 1934), is very thin and the preserved part does not meet the other nerves of the plexus, which are much thicker. However, in view of its orientation, we presume that it took part in the plexus and that the missing part results from incomplete fossilization or from an insufficient contrast on tomograms, leading to segmentation artefacts.

**Figure 4:**
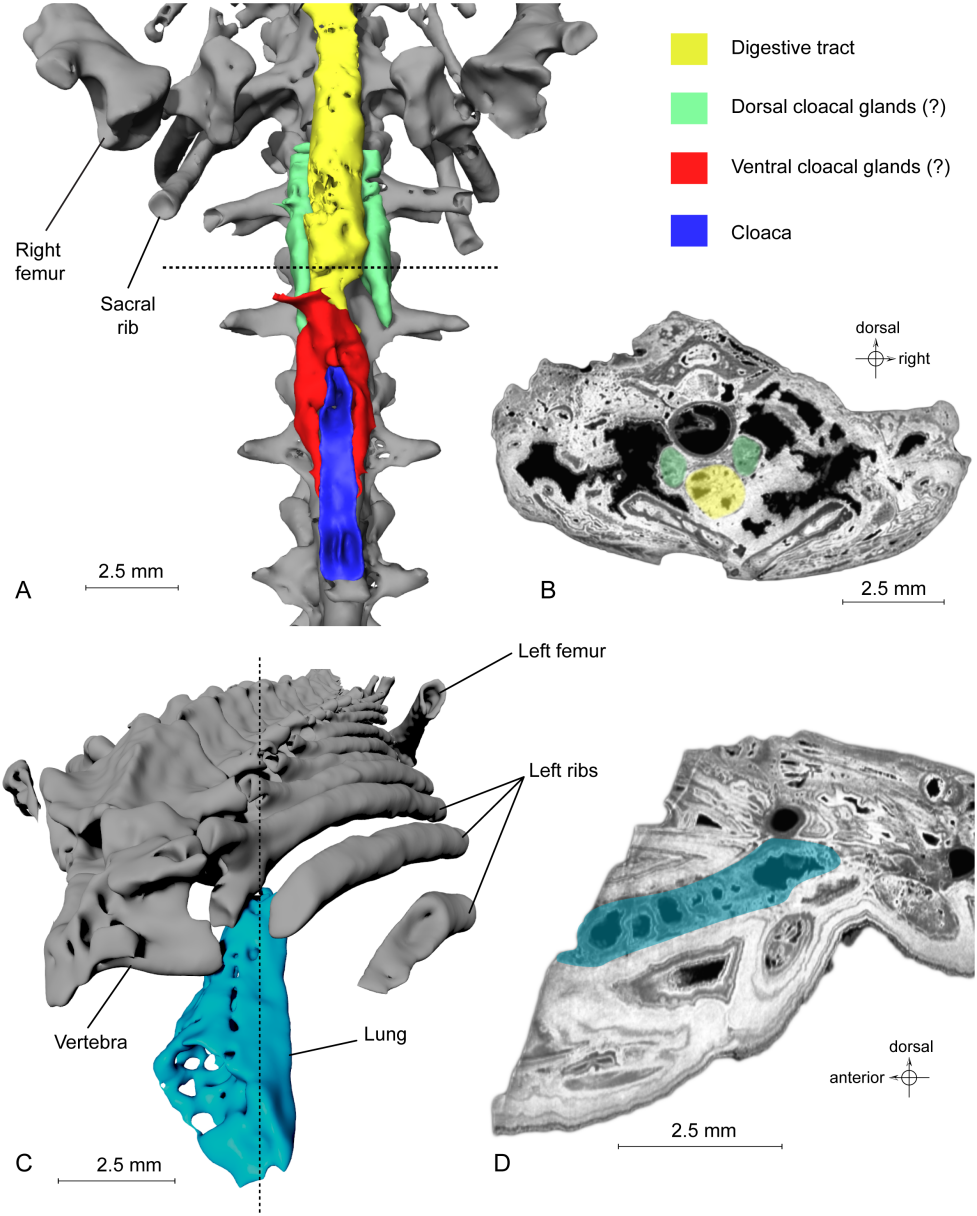
Exceptional preservation of cloacal glands (?) and lung. (A) 3D reconstruction of supposed dorsal and ventral cloacal glands, in ventral view, under the two ischia (not shown). The dorsal cloacal glands are located between the first and second caudosacral vertebrae and the digestive tract (see Fig. 4B). The ventral cloacal glands are located under the digestive tract and anterodorsal to the cloaca. The dotted line represents the position of the virtual section illustrated in Fig. 4B. (B) Virtual section of the pelvic girdle, illustrating the digestive tract and the dorsal cloacal glands, between a caudal vertebra and the two ischia. (C) 3D reconstruction of the incomplete lung (in blue), inside the specimen MNHN.F.QU17755, in oblique anterior view. It is located lateroventrally to the trunk vertebrae, in the anteriormost preserved part of the fossil. The dotted line represents the position of the tomogram illustrated in Fig. 4D. (D) Virtual section of the anteriormost preserved part of MNHN.F.QU17755, showing the inside of the lung in lateral view.

**Digestive system**. The alimentary canal is particularly easy to identify by its circular outline on the tomograms in transverse section. It is visible in most of the specimen length, up to the level of the pelvic girdle. It is very well preserved and its shape in three dimensions leaves little to no doubt about its identification (Figs. 3F–3G). Its diameter is quite variable and no well-defined stomach may be discerned, which is a characteristic of various urodeles (Delsol, Flatin & Exbrayat, 1995).

Here, the content of the digestive system is preserved (Figs. 3C–3E), a very rare and exceptional phenomenon: a few bones are present in the digestive tract, including a small humerus (five mm long) of an undetermined anuran, recognizable by its typical spherical distal articular condyle. Four vertebrae in connection are also present and could belong to that same young anuran.

**Urogenital organ**. Two paired organs are located just posterior to the pelvic girdle: one ventral to the first two caudosacral vertebrae, the other ventral to the second and third caudosacral vertebrae and dorsal to the cloaca (Figs. 4A–4B). Each is comprised of two elongate, fusiform elements situated on both sides of the cloaca. On the specimen, the cloaca is an elongate slit located just posterior to the hind limbs (Figs. 1B, 3F and 4A). These paired organs are approximately five mm long. Both parts of the dorsal most organ, ventral to the first two caudosacral vertebrae, are connected by a plate-like structure that is probably an artefact, given that it was difficult to differentiate it from the surrounding matrix and other elements during segmentation. The two parts of the ventral most organ are also connected, but it is very difficult to tell how, because of low contrast on tomograms. Assuming that these two organs are really paired, i.e., that the plate-like element is an artefact, the elongate parts may represent cloacal glands, the testicles, or the kidneys. In urodeles, testicles and kidneys may be similarly elongated (Delsol, Blond-Fayolle & Flatin, 1995; Gipouloux & Cambar, 1995), but they are located more cranially. These structures are thus more likely to represent dorsal and ventral cloacal glands, but this conclusion must remain tentative because the morphology of these glands in extant urodeles remains poorly described, though some histological descriptions have been published (Sever, 1981; Sever, 1992). According to Francis (1934), the male cloaca is surrounded by ‘a large tubular gland’, which fits the description of the ventral glands preserved here. These glands are not found in females *Salamandra*, which would mean that this fossil specimen was a male.

**Lung**. It was briefly described by Tissier et al. (2016), but a new description is given here, nevertheless. This organ is observable at the anterior part of the specimen, on the left side (Fig. 4C). The anterior portion is missing. The preserved part is triangular in dorsal or ventral view, its tip being directed caudally, and flattened in cross section (Fig. 4D). The section shows a vacuolar structure. Despite the absence of the anterior portion, the position of that organ in the body, ventral to the ribs (in the thoracic region), its shape and its vacuolar internal structure suggest that it is a lung (Francis, 1934; M Laurin, pers. obs., 2014). Within Caudata, the presence of a lung is primitive, but it remains useful to exclude some taxonomic affinities (i.e., within Plethodontidae).

## Discussion

**Ecology.** The presence of anuran bones in the digestive tract of the fossil (Figs. 3C–3E) is evidence of a type of predation that is very rare in urodeles. Preying on frogs was reported in *Amphiuma* (Montaña, Ceneviva-Bastos & Schalk, 2014), a large and especially voracious extant urodele. Another voracious urodele, *Necturus*, has been reported (Hamilton, 1932) to have eaten other urodeles (*Desmognatus* and *Eurycea*), but not frogs. *P. sigei* was relatively small and the swallowed anuran, although small, was likely a metamorphosed individual, as shown by the well-shaped humeral condyle, but not a fully grown adult, as shown by the broad neural canal, assuming that the vertebrae belong to the same individual as the humerus. The straight diaphysis of the humerus and the position of the humeral condyle in line with the diaphysis suggest that the prey was a ranoid. Ranoids were already reported from the Phosphorites (Rage, 1984; Rage, 2016). The length of the humerus (five mm) suggests that the individual measured about 18–20 mm in snout-vent length.

To further investigate the ecology of the animal, we studied the microanatomy of the femur, through a transverse virtual section of the diaphysis on tomograms, and calculated its compactness profile with the software Bone Profiler (Girondot & Laurin, 2003). Without much surprise, both inference models (based on backward elimination and forward selection procedures, respectively) presented by Laurin, Canoville & Quilhac (2009) suggest an amphibious or terrestrial lifestyle (see Supplemental Information). This would suggest that *P. sigei* was not neotenic because all extant neotenic urodeles are strictly aquatic.

**Exceptional preservation.** The three-dimensionally preserved organs described here rank among the oldest known in vertebrates (even though the geological age of the studied fossil could only be determined indirectly). Putative lungs were described from the Devonian *Bothriolepis* (Denison, 1941), but this interpretation has recently been refuted by Goujet (2011). A probable ‘lung’ has also been observed in the actinistian sarcopterygian *Axelrodichthys araripensis* from the Cretaceous (Brito et al., 2010), but it is structurally very different from the regular lung of other vertebrates; it is geologically older than the lung of *Phosphotriton sigei*, but its fossilization is linked to the fact that it was originally mineralized (*in vivo*). The spinal cord, although we have not segmented it and it is not visible on all original virtual sections, is partly preserved. It is, to our knowledge, the only case of three-dimensional fossil preservation of that structure. The spinal cord is quite infrequent in the fossil record. It is known in the tadpoles of the Miocene frog *Rana pueyoi*; in fact, in the latter fossils, McNamara et al. (2010) described more precisely the nerve chord, which is the embryonic antecedent of the spinal cord. In these fossils, the cord is preserved in two dimensions. To our knowledge, the specimen of *Phosphotriton* is the only example of a fossilized nerve plexus in vertebrates. The three-dimensional preservation of the digestive tract documented here is also particularly exceptional. In fossils, this tract is generally two-dimensionally preserved, with even sometimes its content (Dal Sasso & Signore, 1998; McNamara et al., 2010), or the tract content may be preserved without impression of the tract itself (e.g., Piñeiro et al., 2012), but never to our knowledge have a three-dimensional fossilized tract and its content been reported in vertebrates; however, three-dimensional tracts, with perhaps remnants of the content, have recently been described in fossilized arthropods, which also come from the ‘Phosphorites du Quercy’ (Schwermann et al., 2016a). *Phosphotriton* may also be the only case of fossilization of an organ of the urogenital system (likely cloacal glands) among vertebrates (even though our interpretation of this structure is tentative) and it is the first known instance of an extinct salamander taxon and of a putative salamandrid (extinct or not) that fed on an adult anuran. Muscles reported here, on the contrary, are not the oldest known, as they have been reported in *Eastmanosteus calliaspis*, a Late Devonian placoderm (Trinajstic et al., 2007) and in the actinistian sarcopterygian *Wenzia latimerae* from the Late Oxfordian (Clément, 2005), for example.

This case of exceptional preservation is difficult to explain, more specifically as the fossiliferous locality that produced the fossil is unknown. It is suspected, but cannot be demonstrated, that all mummies from the ‘Phosphorites du Quercy’ come from a single, lost locality. It is striking that none of the numerous fossiliferous sites of the Phosphorites du Quercy investigated during the last five decades or so produced ‘mummies’. Equally strange is that none of the mummies pertain to Mammalia, as most skeletal remains found in the Phosphorites du Quercy are mammals. Instead, all belong to ectothermic tetrapods (lissamphibians and snakes; Rage, 2006) and to arthropods (Schwermann et al., 2016a; Schwermann et al., 2016b). Might this result from a taphonomic filter? Was the environment in which these fossils formed (only for the lost locality that yielded mummies) more suitable for lissamphibians and snakes than for mammals? The fact that this locality is now lost prevents us from answering the questions raised above, for now. However, Schwermann et al. (2016a) suggested that such fossils (i.e., arthropods in that case) formed by rapid permineralization of phosphate transported by water that circulated in the fissures and fillings. They suggested that the source of phosphate might have been the numerous bones that accumulated in the fissures. However, another origin of vertebrate mummies deserves consideration. Bats are very numerous in the localities of the Phosphorites (Sigé & Hugueney, 2006) and they likely produced a large amount of guano. Bat guano, which is very rich in phosphate, is known to facilitate preservation in the presence of calcite (Shahack-Gross et al., 2004). Permineralization of soft tissues by phosphorus leading to exceptional preservation was already observed in a few other cases, for embryophytes, arthropods and gastropods (Arena, 2008), ostracod sperm (Matzke-Karasz et al., 2014), and annelids (Wilson et al., 2016). Schwermann et al. (2016a) also showed that air-dried specimens (as can be observed nowadays in lissamphibians after post-mortem desiccation) do not accurately preserve soft tissues. This suggests that dead animals were rapidly buried in the sediment, a prerequisite for phosphatization of soft tissues (Wilson et al., 2016), where they were infiltrated by percolating water and thus permineralized. In any case, given the amazing three-dimensional preservation of soft tissues, we believe that it is appropriate to classify the lost locality of the ‘Phosphorites du Quercy’ that produced the vertebrate mummies (and the locality that yielded the arthropod mummies) as a Fossil Konservat-Lagerstätte.

## Conclusions

The only specimen of *Phosphotriton sigei* represents a peculiar case of exceptional preservation, in which several organs are preserved in three dimensions, in addition to the skeleton: lung, spinal cord, lumbosacral plexus, digestive tract, muscles, and an unidentified urogenital organ. In addition, the alimentary tract contains skeletal remains of a frog, which is a very rare prey for salamanders. Contrary to the above-cited case of arthropods (Schwermann et al., 2016a), we do not believe that the new data on soft anatomy will revolutionize our understanding of lissamphibian evolution, particularly because such characters have played a modest role in phylogenetic studies of lissamphibians. However, these data, such as the presence of a lung, proved critical to place the mummy in the phylogeny, and these data document the oldest known occurrence of anurophagy in urodeles.

##  Supplemental Information

10.7717/peerj.3861/supp-1 Supplemental Information 1 3D model of the skeleton of MNHN.F.QU17755, holotype of *Phosphotriton sigei* File should be open with Adobe Acrobat Reader for 3D content. Unit for measurement is in cm.

10.7717/peerj.3861/supp-2 Supplemental Information 2 Results of Bone Profiler analyses for bone microanatomy of the femur of MNHN.F.QU17755 Blue line of the graph is radial profile; red line is global profile.

## Additional Information and Declarations

### Data Availability

The following information was supplied regarding data availability: The 3D model of the holotype of Phosphotriton sigei, in 3D PDF file format, has been provided as a Supplemental File.

## References

[ref-1] Arena DA (2008). Exceptional preservation of plants and invertebrates by phosphatization, Riversleigh, Australia. Palaios.

[ref-2] Brito PM, Meunier FJ, Clément G, Geffard-Kuriyama D (2010). The histological structure of the calcified lung of the fossil coelacanth *Axelrodichthys araripensis* (Actinistia: Mawsoniidae). Palaeontology.

[ref-3] Clément G (2005). A new coelacanth (Actinistia, Sarcopterygii) from the Jurassic of France, and the question of the closest relative fossil to *Latimeria*. Journal of Vertebrate Paleontology.

[ref-4] Dal Sasso C, Signore M (1998). Exceptional soft-tissue preservation in a theropod dinosaur from Italy. Nature.

[ref-5] Davis BM, Duffy MT, Simpson SB (1989). Bulbospinal and intraspinal connections in normal and regenerated salamander spinal cord. Experimental Neurology.

[ref-6] Delsol M, Blond-Fayolle C, Flatin J, Grassé PP (1995). Appareil génital mâle. Anatomie-histologie, déterminisme du cycle sexuel. Traité de zoologie XIV.

[ref-7] Delsol M, Flatin J, Exbrayat JM, Grassé PP (1995). Le tube digestif des amphibiens adultes. Traité de zoologie XIV.

[ref-8] Denison RH (1941). The soft anatomy of *Bothriolepis*. Journal of Paleontology.

[ref-9] Francis ETB (1934). The anatomy of the salamander.

[ref-10] Gipouloux JD, Cambar R, Grassé PP (1995). Anatomie du rein. Traité de zoologie XIV.

[ref-11] Girondot M, Laurin M (2003). Bone Profiler: a tool to quantify, model, and statistically compare bone-section compactness profiles. Journal of Vertebrate Paleontology.

[ref-12] Goujet D (2011). “Lungs” in Placoderms, a persistent palaeobiological myth related to environmental preconceived interpretations. Comptes-Rendus Palevol.

[ref-13] Hamilton WJ (1932). The food and feeding habits of some eastern salamanders. Copeia.

[ref-14] Laloy F, Rage JC, Evans SE, Boistel R, Lenoir N, Laurin M (2013). A re-interpretation of the eocene anuran *Thaumastosaurus* based on MicroCT examination of a ‘mummified’ specimen. PLOS ONE.

[ref-15] Laurin M, Canoville A, Quilhac A (2009). Use of paleontological and molecular data in supertrees for comparative studies: the example of lissamphibian femoral microanatomy. Journal of Anatomy.

[ref-16] Legendre S, Sigé B, Astruc JG, Bonis L, Crochet JY, Denys C, Godinot M, Hartenberger JL, Lévêque F, Marandat B, Mourer-Chauviré C, Rage JC, Rémy JA, Sudre J, Vianey-Liaud M (1997). Les phosphorites du Quercy: 30 ans de recherche. Bilan et perspectives. Geobios.

[ref-17] Matzke-Karasz R, Neil JV, Smith RJ, Symonová R, Mořkovský L, Archer M, Hand SJ, Cloetens P, Tafforeau P (2014). Subcellular preservation in giant ostracod sperm from an early Miocene cave deposit in Australia. Proceedings of the Royal Society of London B.

[ref-18] McNamara ME, Orr PJ, Kearns SL, Alcala L, Anadon P, Peñalver Mollá E (2010). Exceptionally preserved tadpoles from the Miocene of Libros, Spain: ecomorphological reconstruction and the impact of ontogeny upon taphonomy. Lethaia.

[ref-19] Montaña CG, Ceneviva-Bastos M, Schalk CM (2014). New vertebrate prey for the aquatic salamander *Amphiuma means* (Caudata: Amphiumidae). Herpetology Notes.

[ref-20] Pélissié T, Sigé B (2006). 30 millions d’années de biodiversité dynamique dans le paléokarst du Quercy [30 million years of dynamic biodiversity in the paléokarst of Quercy].

[ref-21] Piñeiro G, Ramos A, Goso C, Scarabino FS, Laurin M (2012). Unusual environmental conditions preserve a Permian mesosaur-bearing Konservat-Lagerstätte from Uruguay. Acta Palaeontologica Polonica.

[ref-22] Rage JC (1984). Are the Ranidae (Anura, Amphibia) known prior to the Oligocene?. Amphibia-Reptilia.

[ref-23] Rage JC (2006). The lower vertebrates from the Eocene and Oligocene of the Phosphorites du Quercy (France): an overview. Strata.

[ref-24] Rage JC (2016). Frogs (Amphibia, Anura) from the Eocene and Oligocene of the Phosphorites du Quercy (France). An overview. Fossil Imprint.

[ref-25] Schwermann AH, Dos Santos Rolo T, Caterino MS, Bechly G, Schmied H, Baumbach T, Van de Kamp T (2016a). Preservation of three-dimensional anatomy in phosphatized fossil arthropods enriches evolutionary inference. eLife.

[ref-26] Schwermann AH, Wuttke M, Dos Santos Rolo T, Caterino MS, Bechly G, Schmied H, Baumbach T, Van de Kamp T (2016b). The fossil insects of the quercy region: a historical review. Entomologie Heute.

[ref-27] Sever DM (1981). Cloacal anatomy of male salamanders in the families Ambystomatidae, Salamandridae, and Plethodontidae. Herpetologica.

[ref-28] Sever DM (1992). Comparative anatomy and phylogeny of the cloacae of salamanders (Amphibia: Caudata). IV. Salamandridae. The Anatomical Record.

[ref-29] Shahack-Gross R, Berna F, Karkanas P, Weiner S (2004). Bat guano and preservation of archaeological remains in cave sites. Journal of Archeological Science.

[ref-30] Sigé B, Hugueney M (2006). Les micromammifères des gisements à phosphate du Quercy (SW France). Strata.

[ref-31] Skutschas PP (2009). Re-evaluation of *Mynbulakia* (Lissamphibia: Caudata) and description of a new salamander genus from the Late Cretaceous of Uzbekistan. Journal of Vertebrate Paleontology.

[ref-32] Skutschas PP, Baleeva NV (2012). The spinal cord supports of vertebrae in the crown-group salamanders (Caudata, Urodela). Journal of Morphology.

[ref-33] Tissier J, Rage J-C, Boistel R, Fernandez V, Pollet N, Garcia G, Laurin M (2016). Synchrotron analysis of a ‘mummified’ salamander (Vertebrata: Caudata) from the Eocene of Quercy, France. Zoological Journal of the Linnean Society.

[ref-34] Trinajstic K, Marshall C, Long J, Bifield K (2007). Exceptional preservation of nerve and muscle tissues in Late Devonian placoderm fish and their evolutionary implications. Biology Letters.

[ref-35] Wake DB, Lawson R (1973). Developmental and adult morphology of the vertebral column in the plethodontid salamander *Eurycea bislineata*, with comments on vertebral evolution in the amphibia. Journal of Morphology.

[ref-36] Wilson P, Parry LA, Winther J, Edgecombe GD (2016). Unveiling biases in soft-tissue phosphatization: extensive preservation of musculature in the Cretaceous (Cenomanian) polychaete *Rollinschaeta myoplena* (Annelida: Amphinomidae). Palaeontology.

[ref-37] Wischnitzer S (1979). Atlas and dissection guide for comparative anatomy.

