# Supplementary material for: Exceptional soft tissues preservation in a mummified frog-eating Eocene salamander
Source: PeerJ. 2017 Oct 3;5:e3861. doi: 10.7717/peerj.3861 (PMC5629955; doi:10.7717/peerj.3861)

3D model of MNHN.F.QU17755, holotype of *Phosphotriton sigei*. Open file with Adobe Acrobat Reader for 3D viewing. File unit is in cm.

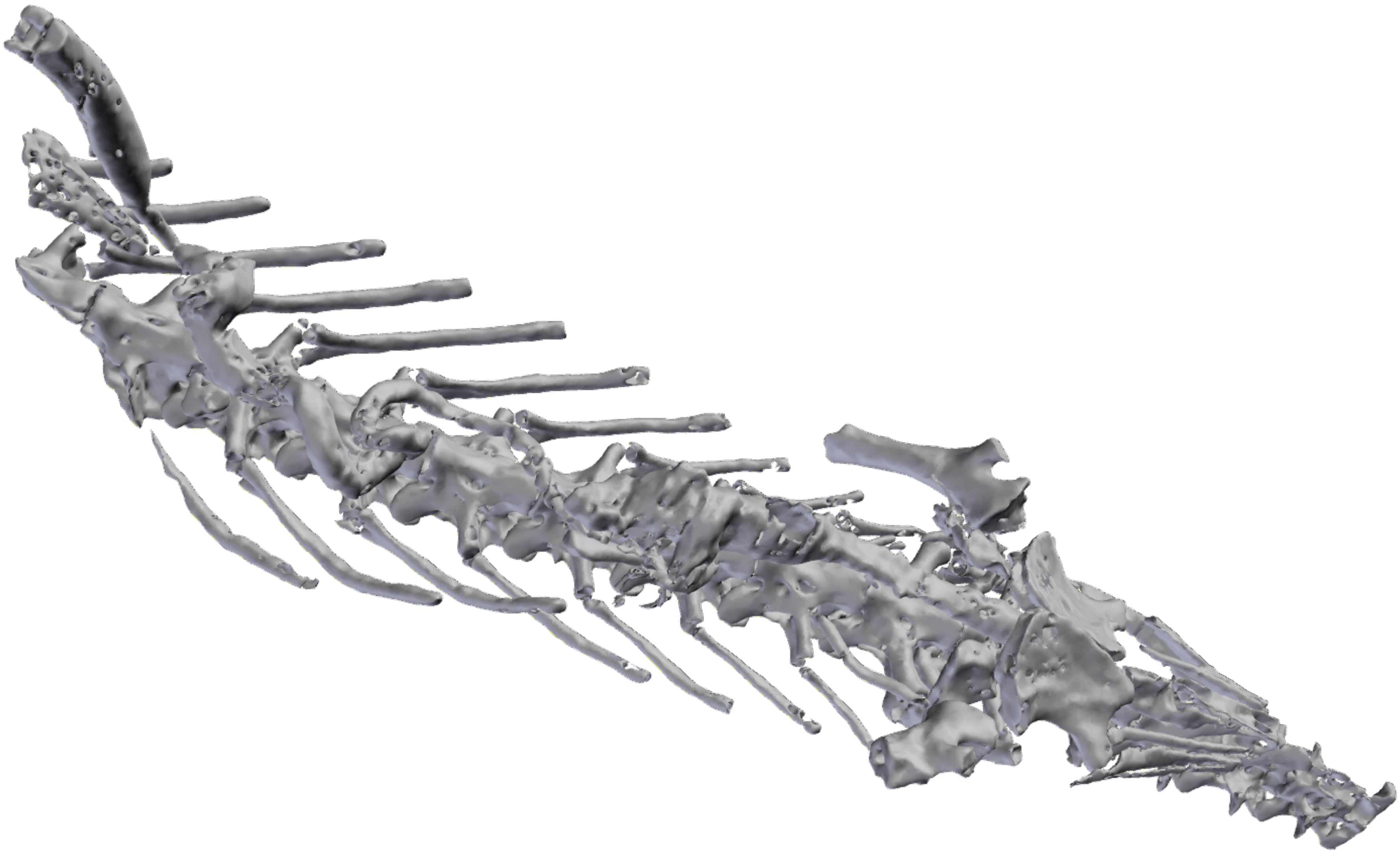

Supplement: Supplemental Information 1 — File should be open with Adobe Acrobat Reader for 3D content. Unit for measurement is in cm. [file peerj-05-3861-s001.pdf]
